# Uev1A facilitates osteosarcoma differentiation by promoting Smurf1-mediated Smad1 ubiquitination and degradation

**DOI:** 10.1038/cddis.2017.366

**Published:** 2017-08-03

**Authors:** Weiwei Zhang, Yuan Zhuang, Yiran Zhang, Xiaoran Yang, Hong Zhang, Guifen Wang, Wanqi Yin, Ruifeng Wang, Zhiling Zhang, Wei Xiao

**Affiliations:** 1 College of Life Sciences, Capital Normal University, Beijing, China; 2 Department of Microbiology and Immunology, University of Saskatchewan, Saskatoon, Saskatchewan, Canada

## Abstract

Malignant bone tumor osteosarcoma (OS) displays high metastasis incidence and poor prognosis. Its stem cell properties could serve to explain tumor recurrence and resistance to conventional treatments. In this study, we identified *UEV1A* as a novel suppressor of OS. Elevated *UEV1A* diminishes stem cell properties of OS cells and drives them to terminal differentiation. Importantly, *UEV1A*-overexpressed OS cells delay proliferation and are more sensitive to chemotherapeutic agents than control cells. Uev1A appears to be involved in the BMP signaling pathway in which it collaborates with a ubiquitin E3 ligase Smurf1 to promote Smad1 degradation in a Ubc13-independent manner. Indeed, Smad1 is identified as a dominant downstream effector of Uev1A, which unravels the mechanism underlying Uev1A-orchestrated tumor suppression in OS. The above findings identify *UEV1A* as a potential OS tumor suppression gene, and shed lights to future OS diagnosis and treatment.

Osteosarcoma (OS) is a malignant bone tumor that displays high metastasis incidence and chemoresistance.^1, 2^ Conventional treatments of OS usually combine surgical resection with radiotherapy or chemotherapy, such as isofosfamide, doxorubicin, cisplatin and methotrexate. However, the prognosis of OS patients is poor, especially for those who present metastasis.^3, 4, 5^ Thus, it is necessary to identify a more efficient therapy to cure OS. Recent studies suggest that OS derives from defect in differentiation of mesenchymal stem cells (MSCs).^6, 7^ MSC differentiation can be divided into several stages including MSCs, committed osteoprogenitor, pro-osteoblast, early osteoblast, mature osteoblast and osteocyte,^8^ which can be characterized by their representative marker gene expression. For example, inhibitors of differentiation (*ID*s) have the highest expression level in MSCs and support high proliferating capability of cells.^9, 10^ During differentiation, the expression of *ID* genes markedly decreases, whereas those of *RUNX2* and *OSTERIX* increase.^11^ Runx2 and Osterix are important osteogenic regulators exhibiting the highest level in the pro-osteoblast stage.^8^ Late markers like osteocalcin (OC) and osteopontin (OPN, SPP1) feature mature osteoblasts and osteocytes.^8^ As block of differentiation leads to gathering of stem cell-like cells that maintain high proliferation capability, it is assumed that defect in any of these MSC differentiation stages may result in OS. These properties of OS cells appear to be similar to those of cancer stem cells (CSCs) with elevated expression of stem cell marker genes.^12, 13^ Although accounting for a small cancer cell population, CSCs seem to orchestrate cancer recurrence and resistance to conventional treatments.^14, 15^ Reduction of CSCs by inducing differentiation or disrupting CSC niche may sensitize cancer cells to chemotherapy or radiotherapy.

Ubiquitin-conjugating enzyme (Ubc) E2 variants (Uevs) are related to Ubc in sequence but do not contain the active Cys residue for ubiquitination.^16, 17^ Uevs specifically interact with Ubc13, which is the only E2 dedicated to mediate K63-linked poly-Ub chain assembly.^18, 19^ Several lines of evidence support a close correlation between *UEV1A* and carcinogenesis, probably because it forms a stable complex with Ubc13 to activate the NF-κB pathway,^20, 21^ which promotes tumorigenesis and metastasis.^22^ Uev1A is negatively correlated with differentiation as its expression is diminished upon differentiation in human colon adenocarcinoma cells.^17^ Interestingly, previous studies show that the NF-*κ*B signaling is involved in controlling osteolineage specification.^23, 24, 25^ Therefore, as a key activator of this pathway, Uev1A could have a potential role in regulating poorly differentiated OS. In this study, we identified Uev1A as a novel OS regulator and found that it works with a E2-E3 complex UbcH5B-Smurf1 to facilitate the ubiquitination and degradation of an OS promoting factor Smad1, which reveals a critical role of Uev1A in promoting osteoblast differentiation and preventing OS. Importantly, *UEV1A*-driven OS differentiation markedly inhibits the ‘stemness’ of OS cells, which confers OS cell sensitivity to chemotherapeutic agents. Hence, enhanced Uev1A activity may serve as a means of treating OS.

## Results

### Uev1A promotes differentiation of OS cells in a Ubc13-independent manner

**Figure 1:**
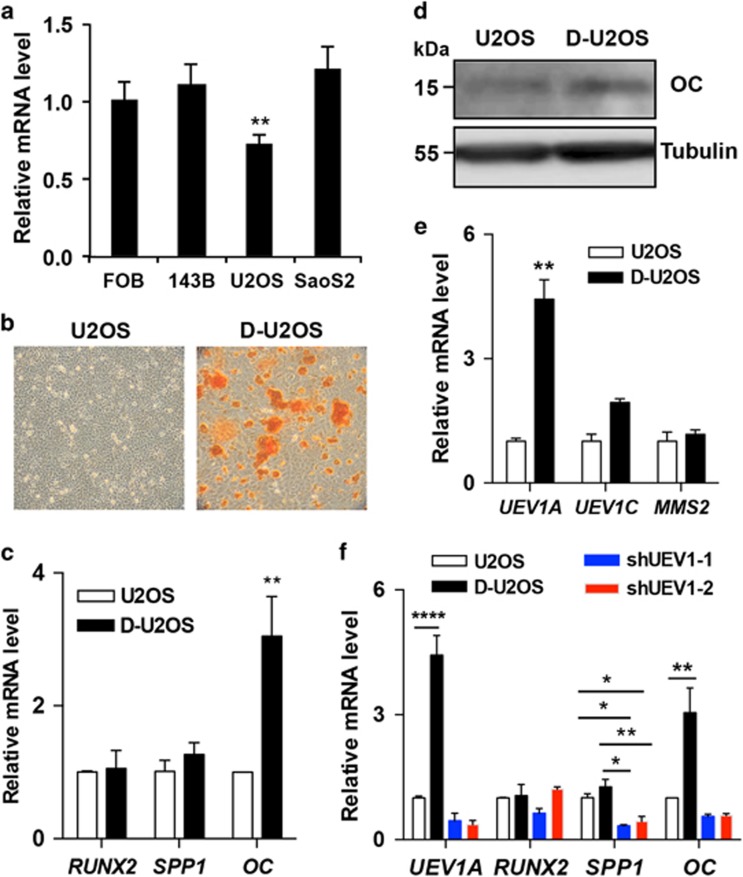
Uev1A fluctuation during OS cell differentiation. (**a**) The expression of Uev1A in multiple OC cell lines. (**b**) Establishment of differentiated OS cells. U2OS cells were induced to differentiate through culturing them in an osteogenic medium for 4 weeks followed by Red S staining. (**c**) Altered expression of differentiation marker genes upon cell differentiation. The gene expression was measured by qRT-PCR. Data are presented as the mean±S.D. (**d**) Alteration of OC protein levels during differentiation. Cell lysates were analyzed by western blot using anti-OC and anti-tubulin antibodies. (**e**) Alteration of *UEV1A* and other *UEV* transcript levels upon cell differentiation. The transcripts of *UEV1A*, *UEV1C* and *MMS2* were analyzed by qRT-PCR. Data are presented as the mean±S.D. (**f**) Reversal of OS differentiation by *UEV1* depletion. ShRNA-mediated *UEV1* knockdown was performed in the differentiated U2OS cells. Two different anti-*UEV1* shRNA sequences were used to reduce off-target effects. Four days after transfection, total RNA was extracted for the qRT-PCR assay. The expression levels of Uev1A and marker genes in D-U2OS and shRNA-transfected D-U2OS cells are normalized to their corresponding values in empty vector-transfected wild-type U2OS cells. Data are the mean±S.D. D-U2OS: differentiated U2OS cells

To investigate the role of Uev1A in regulating OC cell differentiation, first of all, we screened its expression in multiple OC cell lines, including FOB, 143B, U2OS and Saos2 cells. Compared with the normal FOB osteoblast cells, only U2OS cells displayed a significantly reduced expression of Uev1A (Figure 1a). Next, we induced U2OS cells to differentiate through culturing them in osteogenic medium for 4 weeks. A Red S staining assay was used to detect mineralization in osteoblast cells. As expected, an obviously enhanced mineralization signal was detected in the osteogenic medium-cultured cells (Figure 1b), and the *OC* mRNA level was elevated by fourfold (Figure 1c) along with corresponding increase in the OC protein level (Figure 1d), indicating a successful induction of terminal differentiation. Interestingly, although the expression of *UEV1A* did not exhibit obvious change in the early differentiation stage, a fivefold induction of its mRNA was observed in the fully differentiated cells (Supplementary Figure S1; Figure 1e), suggesting that *UEV1A* expression is positively correlated to OC cell differentiation.

The elevated expression appears to be specific for *UEV1A*, as the expression of its splicing variant *UEV1C* or a homologous gene *MMS2* was not markedly altered (Figure 1e). We reasoned that if *UEV1A* has a critical role in the OS differentiation, its depletion should reverse the differentiation process of OS cells. Indeed, expression of two independent *UEV1* short-hairpin RNAs (shRNAs) diminished the osteogenic medium-induced *UEV1A* and reverted the elevated *OC* expression, whereas the *SPP1* expression was reduced by threefold (Figure 1f). As *OC* and *SPP1* are the marker genes of osteocytes,^8^ the inhibition of their expression indicates a failure in U2OS terminal differentiation.

**Figure 2:**
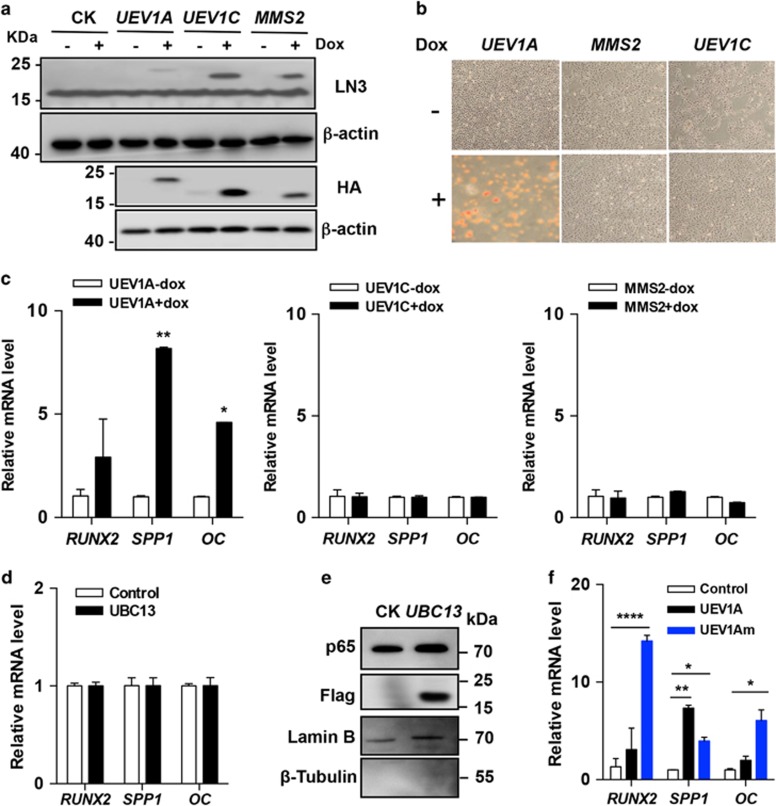
Uev1A promotes U2OS cell differentiation independently of Ubc13. (**a**) Establishment of Dox-inducible *UEV1* and *MMS2* overexpression U2OS stable cell lines. Each gene was fused with a 3xHA tag at the N-terminus before cloning into the mammalian expression vector pcDNA4/TO. Cell lysates were analyzed by western blot (WB) using anti-Uev1/Mms2 (LN3), anti-HA and anti-actin antibodies. (**b**) *UEV1A* overexpression drives U2OS cells to terminal differentiation as indicated by Red S staining. (**c-e**) U2OS cells were transiently transfected with constructs expressing the genes as indicated. Four days after transfection, total RNAs were harvested for the qRT-PCR and WB analyses. Data are presented as the mean±S.D. (**c**) The transcript levels of differentiation markers in *UEV1A*-, *UEV1C*- and *MMS2*-overexpressed stable cell lines as measured by qRT-PCR. (**d**) Overexpression of *UBC13* does not alter the OS differentiation marker gene expression. (**e**) Western blotting showing p65 nuclear translocation upon *UBC13* overexpression. (**f**) A Uev1A mutant (Uev1Am) incapable of interacting with Ubc13 causes stronger differentiation marker gene expression than wild-type Uev1A

To further explore the role of *UEV1A* in OS differentiation, we established Dox-inducible stable U2OS cell lines that expressed ectopic *UEV1A* fused with an HA tag (Figure 2a). In parallel, stable cell lines expressing *UEV1C* or *MMS2* were also generated to serve as controls. Owing to the high degree of similarity in sequence among Uev1A, Uev1C and Mms2, our homemade monoclonal antibody LN3 raised against Uev1A could also detect Uev1C and Mms2 (Figure 2a). Upon Dox-induced *UEV1A* overexpression, mineralization signals were detected in *UEV1A-*overexpressed U2OS cells but not in *UEV1C* or *MMS2*-overexpressed cells (Figure 2b). Meanwhile, the expression levels of late marker genes *OC* and *SPP1* were markedly elevated, along with a moderate increase in *RUNX2* expression (Figure 2c, left panel), indicating that majority of them were terminally differentiated. In sharp contrast, neither *UEV1C* nor *MMS2* overexpression altered the above differentiation marker gene expression (Figure 2c, middle and right panels). These findings collectively demonstrate the role of *UEV1A* in promoting OS differentiation.

Uevs (Uev1 and Mms2) lack the active site Cys residue found in Ubcs and their only known activity to date is a cofactor required for Ubc13-catalyzed Lys63 poly-Ub chain formation.^18, 19^ Surprisingly, Dox-induced *UBC13* overexpression failed to elevate the expression of differentiation markers (Figure 2d), although it was able to strongly enhance nuclear translocation of p65 as expected (Supplementary Figures S2a and b). To further address whether *UEV1A*-induced OC cell differentiation is dependent on Ubc13, we made a Uev1A-F38E (labeled as Uev1Am) mutation that abolishes its physical interaction with Ubc13^26^ (Figure S2c). Overexpression of *UEV1Am* (Supplementary Figure S2d) resulted in an even stronger differentiation marker expression than that of *UEV1A* (Figure 2e), confirming that Uev1A promotes U2OS differentiation in an Ubc13-independent manner.

### *UEV1A* overexpression relieves BMP-induced inhibition of differentiation

**Figure 3:**
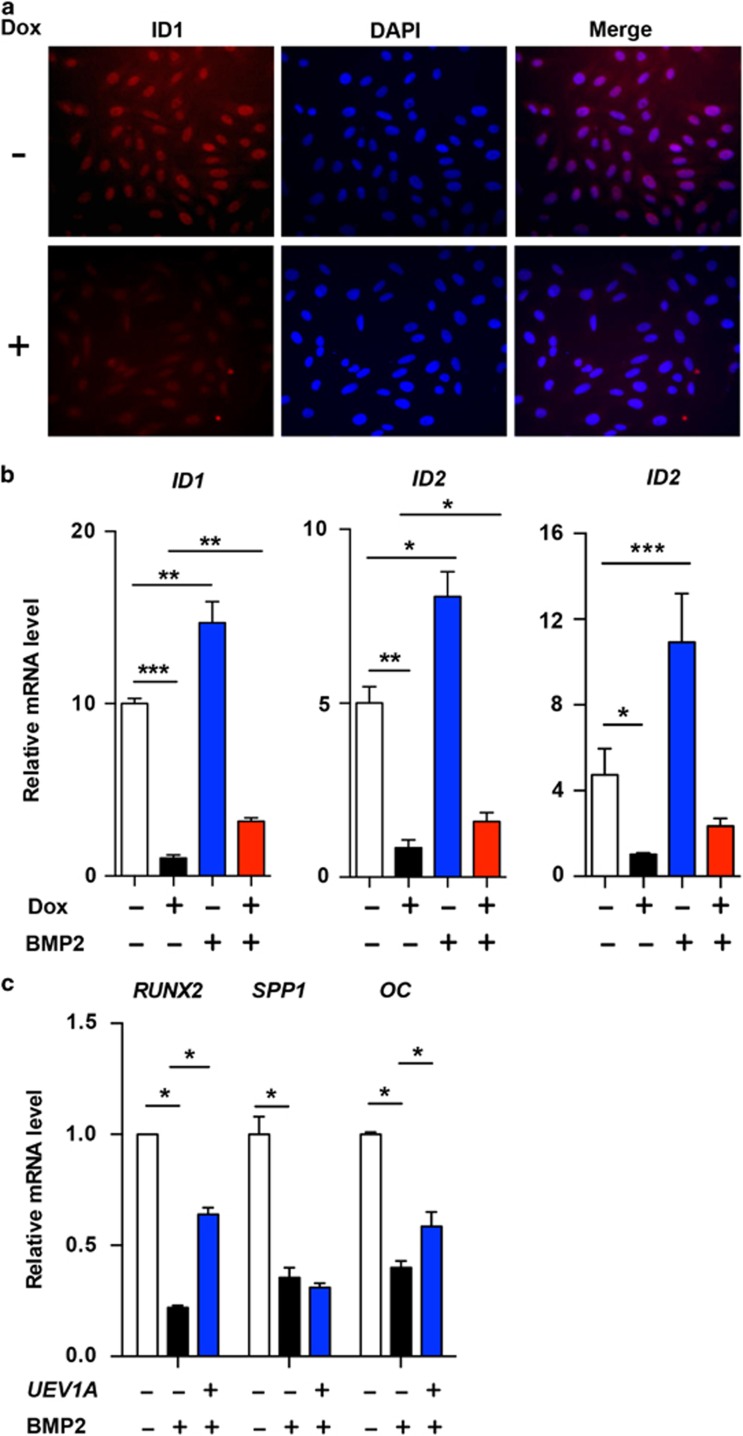
*UEV1A* overexpression antagonizes BMP2 for OS cell differentiation. (**a**) *UEV1A* overexpression suppresses *ID1* as judged by ICC. Representative images of several independent experiments are presented. (**b**) *UEV1A* overexpression inhibits BMP-induced expression of *ID* genes. Stable U2OS transfectants harboring Dox-inducible *UEV1A* in the presence or absence of Dox was untreated or treated with BMP2 for 4 weeks. Total RNAs were harvested for qRT-PCR analysis. (**c**) *UEV1A* overexpression inhibits BMP-repressed differentiation marker genes. The experiment procedure was as described in (**b**)

It has been well established that bone morphogenesis proteins (BMPs) inhibit osteoblast differentiation through transcriptional activation of *ID*s.^9, 10, 27^ As *UEV1A* promotes OS cell differentiation, it would be of interest to see its effects on BMP-induced differentiation block. An ICC assay (Figure 3a) and qRT-PCR (Figure 3b) confirmed that *UEV1A* overexpression inhibited the expression of *ID* genes. As expected,^8^ treatment of U2OS cells with BMP2 induced the *ID* gene expression while repressed *RUNX2*, *OC* and *SPP1* (Figure 3c). Under the above experimental conditions, ectopic expression of *UEV1A* diminished the effect of BMP2 on *ID* genes (Figure 3b) and meanwhile partially relived the inhibitory effect of BMP2 on *RUNX2* and *OC* (Figure 3c). We conclude from the above observations that Uev1A promotes OS differentiation by serving as a negative regulator of the BMP pathway.

### *UEV1A* overexpression suppresses oncogene expression and inhibits the CSC status of OS cells

**Figure 4:**
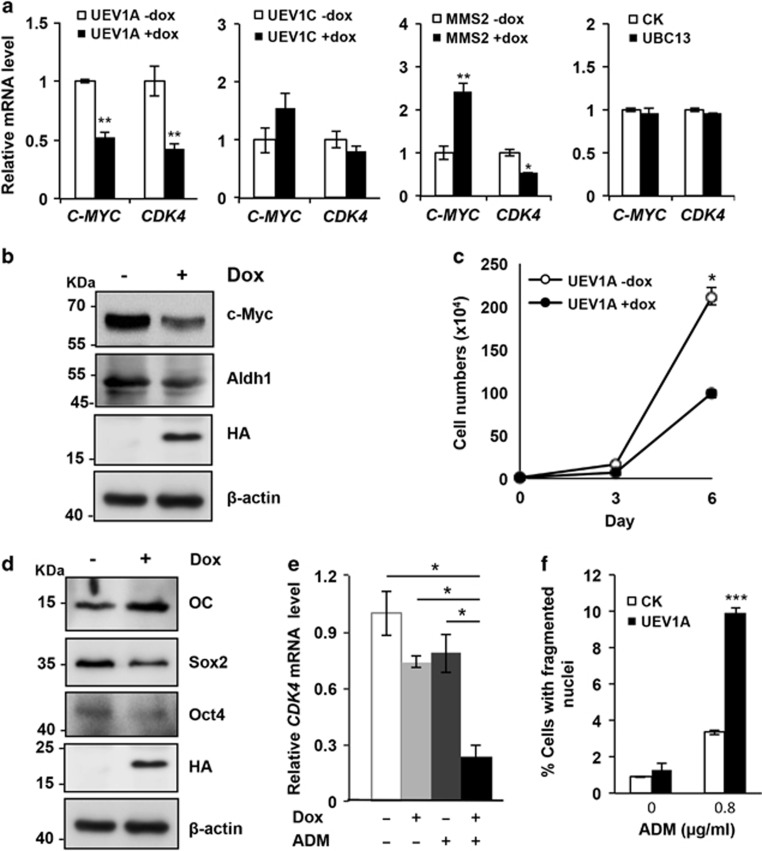
*UEV1A* overexpression inhibits OS-related proto-oncogene and stem cell marker gene expression and cell proliferation. (**a**) Effects of ectopic *UEV* gene expression on the expression of OS-related proto-oncogenes. Total RNA was extracted from control and *UEV*-overexpressed cells followed by qRT-PCR analysis. Data are presented as the mean±S.D. (**b**) WB analysis of c-Myc and Aldh1 levels. (**c**) *UEV1A* overexpression reduces U2OS cell proliferation. After stably transfected U2OS cells were treated with Dox for 4 weeks, collected cells were seeded in the 6D plate with fresh Dox-containing medium and incubated for the indicated period before cell counting. The Dox- population followed the same treatment except adding Dox. (**d**) Ectopic *UEV1A* expression reduces cellular Sox2 and Oct4 levels as revealed by WB. (**e**) Synergistic effects of ectopic *UEV1A* expression and ADM treatment on *CDK4* expression. Total RNAs were extracted for the qRT-PCR analysis and results are presented as mean±S.D. (**f**) *UEV1A* overexpression sensitizes U2OS cells to the anticancer agent ADM. The *UEV1A-*transfected cells were untreated or treated with 0.8 *μ*g/ml ADM for 48 h. *UEV1A*-transfected U2OS cells without Dox induction were used as a control

As BMP treatment has been reported to enhance OS tumor growth,^8^ we speculated that Uev1A could serve as an OS inhibitor through promoting OS differentiation. As the first step toward understanding the role of *UEV1A* in tumor inhibition, we examined the effects of *UEV1A* overexpression on several oncogenes known to be highly expressed in OS cells.^28^ Compared with the control, Dox-induced *UEV1A* reduced *MYC* and *CDK4* expression by >50% (Figures 4a and b and Supplementary Figure S3a) and this effect appears to be specific for *UEV1A*, as ectopic expression of *UEV1C*, *MMS2* or *UBC13* did not cause such consistent reduction (Figure 4a). We also screened other OS-related oncogenes and confirmed the role of *UEV1A* in repressing their expression (Supplementary Figure S3b). The activities of C-Myc and aldehyde dehydrogenase 1 (Aldh1) have been used to define enhanced tumorigenesis and stem cell properties of cancers.^29, 30^ Upon *UEV1A* induction, cellular C-Myc and Aldh1 levels were also markedly reduced (Figure 4b and Supplementary Figures S3a and c). To further explore the biological role of Uev1A in OS, we measured cell proliferation upon *UEV1A* overexpression and found that it markedly decreased tumor cell growth (Figure 4c).

Expression of stem cell marker genes has been taken as evidence of CSCs in OS.^12, 13^ Upon Dox induction of *UEV1A* expression, not only was tumorigenic marker gene expression decreased, the cellular protein levels of Oct4 and Sox2, two classic stem cell markers, were also reduced (Figure 4d and Supplementary Figures S3d and e). Elevated expression of stem cell markers has been taken to explain why OS is refractory to chemotherapy.^1, 2^ To examine whether *UEV1A* overexpression can sensitize OS cells to chemotherapy, we treated U2OS cells with low doses of an anticancer drug adriamycin (ADM) in the presence or absence of ectopic *UEV1A* expression and examined their effects on *CDK4* expression as an indicator of cell cycle progression.^31, 32^ Although 1 *μ*g/ml ADM treatment or *UEV1A* overexpression alone reduced the *CDK4* transcript level by approximately 20%, the combined treatments resulted in a synergistic reduction of *CDK4* expression by 80% (Figure 4e and Supplementary Figure S3f). Although overexpression of *UEV1A* alone had little effect on cell death, it markedly enhanced low-dose ADM-induced cell death from <3 to 10% (Figure 4f), indicating that *UEV1A* expression can indeed sensitize OS cells to effective chemotherapy. We conclude from the above observations that Uev1A promotes OS differentiation, inhibits the stem cell properties of OS and sensitizes OS cells to chemotherapy.

### Uev1A promotes Smurf1-mediated Smad1 degradation

**Figure 5:**
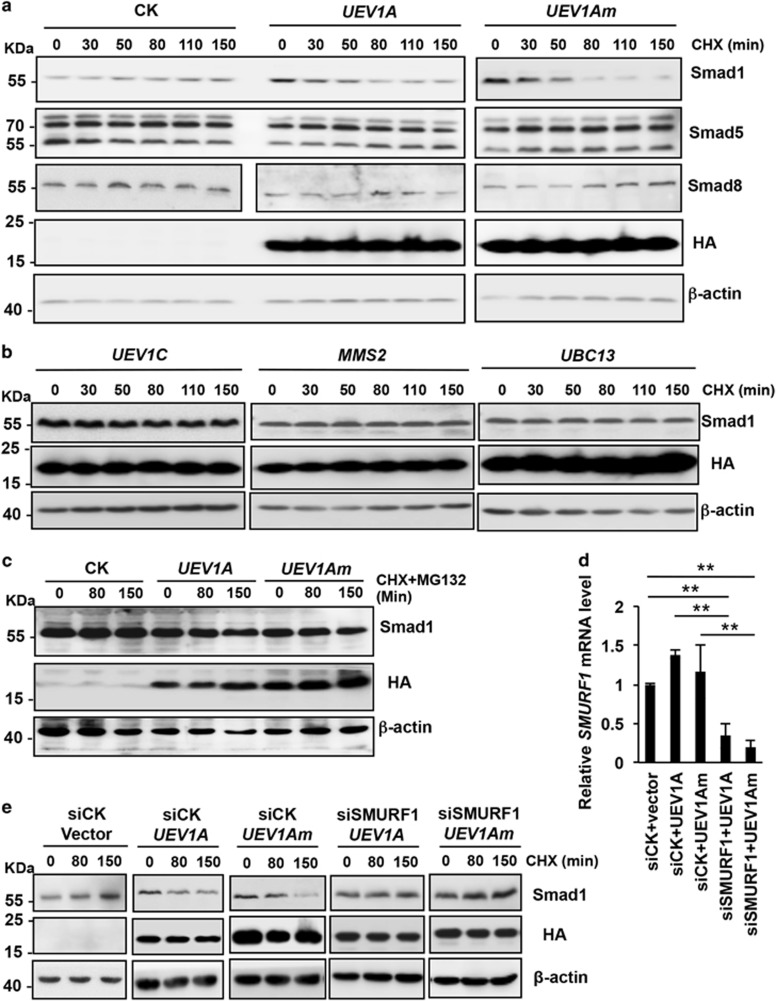
WB analyses of Smad1 stability in the presence of *de novo* protein inhibitor CHX. (**a-e**) U2OS cells were transfected with constructs expressing HA-tagged *UEV*s. Four days after transfection, the cells were treated with 10 *μ*g/ml CHX for the indicated time. Equal amounts of cell extracts were subjected to SDS-PAGE. The related protein levels were examined by WB against indicated antibodies. (**a**) Ectopic expression of either *UEV1A* or *UEV1Am* reduces the cellular Smad1 level, but not Smad5 or Smad8. (**b**) Ectopic expression of *UEV1C*, *MMS2* or *UBC13* does not affect Smad1 stability. (**c**) Smad1 is destabilized by the ubiquitination-26S proteasome degradation system, as treatment of cells with a 26S proteasome inhibitor prevents Uev1A-induced Smad1 degradation. (**d**) Depletion of cellular Smurf1 by siRNA. The relative *SMURF1* transcript level was measured by qRT-PCR following siRNA treatment. (**e**) Uev1A-mediated Smad1 degradation depends on Smurf1. siRNA against *SMURF1* was transfected into the HA-tagged *UEV1A*- or *mUEV1A*-overexpressed U2OS cells. Three days after transfection, the cells were treated with 10 *μ*g/ml CHX for the indicated time points. Equal amount of cell extracts was subjected to SDS-PAGE and WB analysis

BMP signal transduction requires receptor-regulated Smads (R-Smads, including Smad1, 5 and 8), which translocate into the nucleus as transcriptional regulators.^33, 34, 35^ As the activity of R-Smads is primarily regulated by the ubiquitination-26S proteasome system,^36^ we examined cellular Smad levels in response to *UEV1A* expression in the presence of cycloheximide (CHX) that inhibits *de novo* protein synthesis. *UEV1A* overexpression specifically promotes Smad1 but not Smad5 or Smad8 (Figure 5a) degradation over time. Under the same experimental conditions, neither *UEV1C* nor *MMS2* promoted Smad1 degradation (Figure 5b). Notably, overexpression of *UEV1Am* also promotes Smad1 degradation at a rate faster than that of *UEV1A* (Figure 5a), whereas *UBC13* has no such an effect (Figure 5b), consistent with a notion that Uev1A targets Smad1 degradation in a Ubc13-independent manner.

Although Ubc13 is not involved in the Smad1 degradation, the Uev1A-induced Smad1 degradation is mediated by the ubiquitination-26S proteasome system, as treatment of *UEV1A* or *UEV1Am* expressed cells with a 26S proteasome inhibitor MG132 prevented Smad1 degradation (Figure 5c). Smurf1, a HECT-containing E3, is known to trigger ubiquitination of Smad1, 5, 8 and destines them for proteasome-mediated degradation.^37^ To establish a relationship between Uev1A and Smurf1 in the Smad1 degradation, we first confirmed the role of Smurf1 in degrading Smad1 similar to that of *UEV1A* overexpression (Supplementary Figure S4a). Importantly, when Smurf1 was depleted by siRNA in *UEV1A*- or *UEV1Am*-overexpressed U2OS cells (Figure 5d), Smad1 was no longer degraded over time (Figure 5e), indicating that Smurf1 is absolutely required to mediate Uev1A-induced Smad1 degradation and hence Uev1A and Smurf1 belong to the same signaling pathway.

### Uev1A enhances UbcH5B-Smurf1 ubiquitination of Smad1 through physical interaction with Smurf1

**Figure 6:**
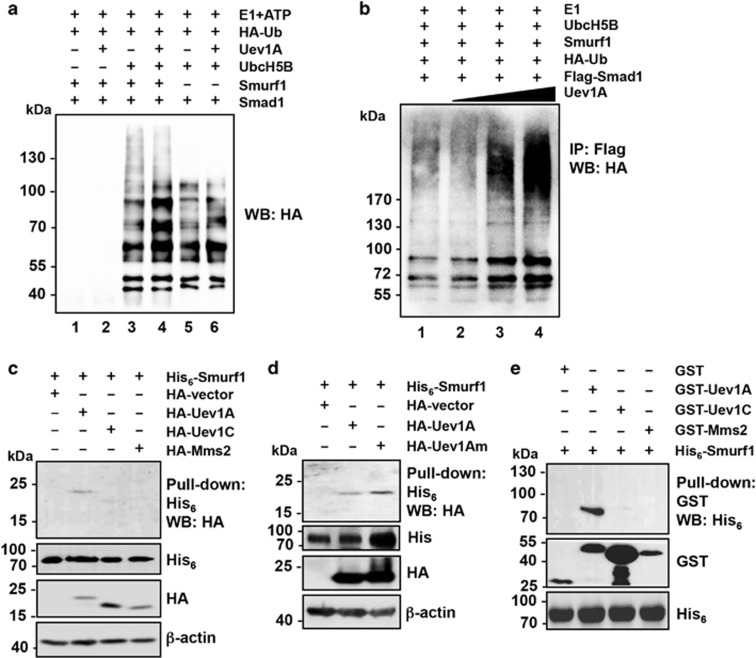
Uev1A interacts with Smurf1 and promotes UbcH5B-Smurf1 ubiquitination of Smad1. (**a**) Uev1A can enhance Smad1 polyubiquitination by UbcH5B-Smurf1 in an *in vitro* ubiquitination assay. Purified components as indicated on the top panel were added in an ubiquitination assay reaction, which was stopped and the products were separated by SDS-PAGE and probed against an HA antibody. (**b**) *In vitro* ubiquitination assay showing the critical role of Uev1A in the process of Smurf1-mediated Smad1 polyubiquitination. Different amounts (10, 20 and 40 nM) of purified Uev1A proteins were used for the reaction. Experimental conditions were as described and the reaction products were immunoprecipitated by anti-Flag beads before WB. (**c**) A His_6_ pull-down assay showing the interaction between Smurf1 and Uev1A. His_6_-tagged Smurf1 protein was purified from bacterial cells and bound to the Nickel beads. Whole-cell extracts from HA-tagged *UEV*-expressing U2OS cells were mixed and incubated with the beads. The elution was subjected to WB by using anti-HA and anti-actin antibodies. Anti-His_6_ antibody was used to show the equal amount of Smurf1 protein was used for the pull-down assay. (**d**) Both Uev1A and Uev1Am were co-purified with His_6_-Smurf1. Experimental conditions were as described in **c**. (**e**) A GST pull-down assay showing the direct interaction between Uev1A and Smurf1. GST-tagged Uevs were purified and bound to the GST affinity beads. His_6_-tagged Smurf1 was purified and mixed with the beads. Elution was further subjected to western blotting assay by using anti-GST and anti-His_6_ antibodies

The above observations suggest that Smurf1 acts as a E3 to mediate Uev1A-promoted OS differentiation and tumor growth. Indeed, in a reconstituted ubiquitination assay, Uev1A itself could not serve as an E2 to mediate Smad1 ubiquitination (Figure 6a, lane 2); however, when UbcH5B, a known Smurf1-interacting E2,^38^ was added along with Smurf1, smear bands characteristic of Smad1 polyubiquitination were observed (lane 3). Moreover, Uev1A markedly increased the intensity of the polyubiquitination chains in a Smurf1-dependent manner (Figure 6a, lanes 4, 5 and 6). In addition, gradually increasing amounts of Uev1a in the reaction resulted in correspondingly enhanced signal of ubiquitinated Smad1 (Figure 6b). Collectively, we concluded that Uev1A is a cofactor of the E2-E3 complex UecH5B-Smurf1 that promotes Smad1 ubiquitination and subsequent degradation, leading to OS differentiation and inhibition of tumor growth.

Uev1A and Mms2 belong to a family of Ubc-E2 variants^39^ known to function as a cofactor of Ubc13.^19, 21^ We first asked in the above ubiquitination reaction, whether Uev1A also functions as a cofactor of UbcH5B. Under conditions of Uev1A-Ubc13 interaction, Uev1A does not appear to bind UbcH5B (Supplementary Figure S4b). We then asked whether Uev1A could serve as a cofactor for Smurf1. In a His_6_-affinity pull-down assay, His_6_-tagged Smurf1 interacted with HA-tagged Uev1A, but not Uev1C or Mms2, from total cell extract (Figure 6c). This interaction does not appear to require Ubc13, as HA-Uev1Am was also co-purified with His_6_-Smurf1, and the stronger signal of Uev1Am than Uev1A (Figure 6d) was either because of an increased expression level of *UEV1Am* over *UEV1A* or the fact that Uev1Am does not bind to Ubc13 for other cellular functions. To address whether Uev1A–Smurf1 interaction is direct, a pull-down assay was performed using bacterially purified proteins. As shown in Figure 6e, GST-tagged Uev1A, but not GST-Uev1C or GST-Mms2, was able to pull-down His_6_-Smurf1, confirming the direct interaction between Uev1A and Smurf1. To further confirm the involvement of Smurf1 and UbcH5B in the differentiation of U2OS cells, we overexpressed *SMURF1* or *UBCH5B*, or depleted cellular Smad1 by siRNA. In all three cases, significant elevation of some OC differentiation marker gene expression was observed (Supplementary Figures S4c-h).

To ask whether the above-observed effects are a general phenomenon in OS cells, we repeated several critical experiments in another OS cell line SaoS2. Overexpression of *UEV1A* caused Smad1 degradation in SaoS2 cells (Supplementary Figure S5a). Similarly, overexpression of *UEV1A, UEV1Am* (Supplementary Figure S5b) or *UBCH5B* (Supplementary Figures S5c and d) induced differentiation marker genes in SaoS2 cells, indicating that this signaling pathway is conserved in OS cells.

## Discussion

**Figure 7:**
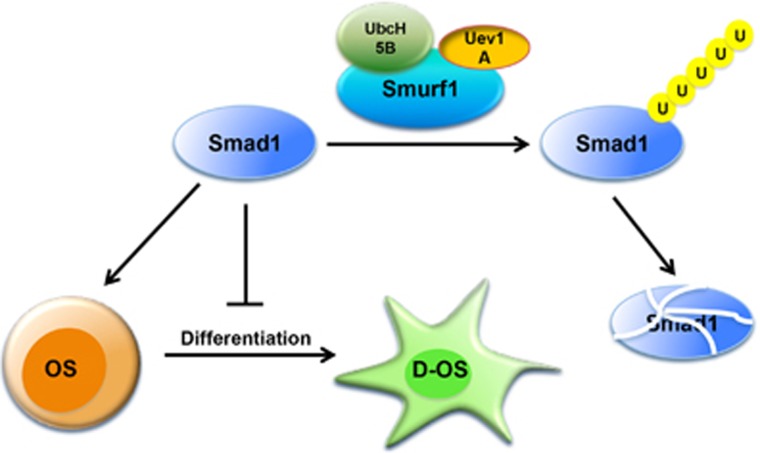
A working model for the role of Uev1A in promoting OS differentiation and thus repressing tumor growth. Uev1A cooperated with UbcH5B and Smurf1 to mediate Smad1 polyubiquitination and its subsequent degradation. This activity drives OS cells to terminal differentiation, which inhibits tumor cell proliferation and sensitizes OS cells to chemotherapy

In summary, one of the key findings in this study is Uev1A-mediated regulation of the BMP pathway where it cooperates with a novel E2-E3 partner UbcH5B-Smurf1 to control cell differentiation. A working model based on findings in this study and previous reports is depicted in Figure 7. This study reveals a critical role of Uev1A in promoting osteoblast differentiation and preventing OS. It also demonstrates that the enhanced Uev1A activity may serve as a means of treating OS. Before this report, eukaryotic Uevs (including Mms2 and Uev1) are only known to function as a partner of Ubc13 to promote K63-linked polyubiquitination.^18, 19^ Ubc13-Uev heterodimers have been attributed to different biological functions, in which Uevs confer certain selectivity.^21^ Surprisingly, in this study Uev1A-mediated Smad1 polyubiquitination is independent of Ubc13, consistent with a notion that Smad1 polyubiquitination leads to its degradation by the 26S proteasome,^36, 40^ whereas Ubc13-Uev mediated K63-polyubiquitination often does not degrade target proteins but alters its cellular function.^41^

Uevs bind to Ubc13 via a unique key-keyhole structure only found in Ubc13s across the entire eukaryotic kingdom.^26^ Apparently this interaction does not apply to Uev1A and UbcH5B. This study reveals that Uev1A directly binds Smurf1 *in vitro* and *in vivo,* and this physical interaction must require its N-terminus, as neither Uev1C nor Mms2 can bind Smurf1. The cognate E3s for Ubc13-Uev to date include RING finger proteins TRAF2/6, TRIM5, budding yeast Rad5 and its mammalian homologs SHPRH and HLTF, and a U-box-containing CHIP, all of which directly interact with Ubc13 and are involved in K63-linked polyubiquitination. In contrast, Smurf1 is a HECT domain-containing E3 involved in regulating the BMP pathway through ubiquitination of Smads.^42^ Although both CHIP and Smurf1 are capable of promoting Smad protein degradation through ubiquitination, CHIP-mediated regulation appears to be independent of BMP stimulation, whereas Smurf1 is tightly controlled by the BMP signaling.^37, 43, 44^

Another striking finding in this study is that Uev1A serves as a novel OS repressor. It is capable of suppressing OS-related oncogenes, reducing the stem cell property and sensitizing OS cells to chemotherapy. These findings collectively point Uev1A as a promising clinic target for OS therapy. This is in sharp contrast to generally conceived roles of Uev1A in tumorigenesis, as previous studies assign *UEV1A* as a putative proto-oncogene.^22, 45^ This is primarily because the Ubc13–Uev1A complex is required for the innate immunity by sensing and activating NF-κB^20, 46, 47^ and possibly other signaling pathways,^48^ and a constitutive *UEV1A* overexpression has been associated with tumorigenesis in several types of cancers. Of particular note is a previous report implying negative correlation between the Uev1 level and colon carcinoma cell differentiation.^17^ We envisage that the biological function of Uev1A should be determined by signaling pathways that it regulates in the given tissue. In this study, Uev1A-promoted OS cell differentiation and the consequent tumor suppression is through inhibiting the unique BMP pathway in OS cells in a Ubc13-independent manner. Hence, it will be of great interest to explore roles of Uev1A in differentiation in other types of cancers.

## Materials and methods

### Cell culture

Human U2OS cells (ATCC, Manassas, VA, USA) were cultured at 37 °C with 5% CO_2_. The cells were maintained in Dulbecco’s modified Eagle's medium (DMEM; GIBCO, GrandIsland, NY, USA), supplemented with 10% fetal bovine serum (FBS; GIBCO) and 5000 units/ml penicillin/streptomycin (Chemicon, Belmopán, Belize, USA).

### Osteogenic differentiation

Seed U2OS cells at the density of 5 × 10^4^ cells/cm^2^ in culture plates with DMEM supplemented with 10% FBS. After 24 h, the culture medium was changed with osteogenic media (DMEM supplemented with 10% FBS, 0.1 *μ*M dexamethasone (Sigma, St. Louis, MO, USA), 10 mM *β*-glycerophosphate (Sigma) and 50 *μ*M ascorbate phosphate (Sigma)) to induce U2OS cell differentiation. The culture medium was changed every other day.

### Protein extraction and western blotting

Total protein was extracted by lysing cells with the whole cell extraction buffer (50 mM Tris-HCl, 150 mM NaCl, 1% NP40, 10% glycerol, 1 mM EDTA and 1 mM PMSF). Thirty micrograms of the total protein was separated by SDS-PAGE and transferred to PVDF membrane. The membrane was blocked with 5% milk and probed with specific primary and secondary antibodies. The blots were developed with ECL Advance Western Blotting Detection Kit (Amersham, Sweden). The following antibodies were used: anti-OC (Santa Cruz, Dallas, TX, USA, sc-18319), anti-Smad1 (Cell Signaling, Danvers, MA, USA, 9743 S), anti-Smad5 (Abcam, Cambridge, MA, USA, ab13724), anti-Smad8 (Santa Cruz, sc-11393), anti-Oct4 (Santa Cruz, sc-8628), anti-Sox2 (Santa Cruz, sc-17320), anti-HA (Santa Cruz, sc-7392), anti-Flag (Sigma, F1804) and anti-*β*-actin. A monoclonal antibody LN3 was made in-house by using purified Uev1A as an antigen, and its epitope recognition region was mapped to amino acids 31–44 with a sequence GVKVPRNFRLLEEG, which is identical to that in Uev1C and Mms2. Hence, LN3 recognizes all three Uevs in this study.

### His6 and GST pull-down assays

For the His pull-down assay, purified His6-tagged Smurf1 fusion protein was pre-cleared with immobilized nickel agarose beads (GE Healthcare, Pittsburg, PA, USA) for 1 h and incubated with the cell extract overexpressing HA-tagged Uev1A, Uev1A-F38E (mUev1A), Uev1C or Mms2 overnight at 4 °C. Protein-bound beads were washed five times with a lysis buffer and eluted in an SDS-PAGE sample buffer. Eluted proteins were analyzed by immunoblotting. For the GST pull-down assay, purified GST-tagged Uev1A, Uev1C or Mms2 fusion proteins were pre-cleared with GST beads (GE Healthcare, 17-0756-0) for 1 h and incubated with purified His6-tagged SMURF1 overnight at 4 °C. Protein-bound GST beads were washed four times with the lysis buffer and eluted in the SDS-PAGE sample buffer. Eluted proteins were analyzed by immunoblotting.

### Immunocytochemistry

Cells were fixed with 4% formaldehyde for 30 min, washed four times over 30 min with PBS plus 0.25 g Tween 20 (PBST) and blocked with 5% horse serum in PBST. Primary anti-Uev1A (homemade), anti-Oct4 (Santa Cruz, SC-8628 1:250), anti-Sox2 (Santa Cruz sc-17320, Fremont, CA, USA, 1:250), anti-C-Myc (Calbiochem OP10, Belmopán, Belize, USA, 1:400), anti-CDK4 (Neomarkers MS-469, Belmopán, Belize, USA, 1:200) and anti-ALDH1 (Abcam ab-6192, 1:500) antibodies were applied in blocking solution for 1 h. After washing in PBST, coverslips were incubated with Alexa546-conjugated anti-mouse (Molecular Probes, 1:3000) and Alexa488-conjugated anti goat (Molecular Probes, Waltham, MA, USA, 1:2000) secondary antibodies for 20 min in the presence of DAPI (2 *μ*g/ml) for 20 min before washing again with PBST and mounting. Images were captured with a 20x oil emersion objective lens, and all red and green images were adjusted identically in order to generate the merge images.

To assess NF-κB activation, cells grown in a slide were transfected with pCMV-3xFlag- UBC13 or the empty vector. After incubation for 48 h, cells were fixed in 4% paraformaldehyde solution (Affymetrix, Waltham, MA, USA) for 20 min at room temperature, permeabilized with 0.4% Triton X-100, blocked in 5% FBS and then incubated with mouse anti-Flag (Sigma, F1804, 1:1000) and rabbit against the p65 subunit of NF-κB (Santa Cruz, sc-372, 1:1000) antibodies overnight at 4 °C. Only Flag-positive cells were counted for the nuclear enrichment of p65.

### RNA isolation, reverse transcription and real-time RT-PCR (qRT-PCR) analysis

Total RNA was extracted using Trizol reagent (Invitrogen, Grand Island, NY, USA). cDNA synthesis was performed with 500 ng of total RNA using RevertAid First Strand cDNA Synthesis kit (Fermentas, Waltham, MA, USA) according to the manufacturer’s instructions. Endogenous mRNA levels were measured by qRT-PCR analysis based on SYBR Green detection (Fermentas) with the Bio-Rad (Hercules, CA, USA) real-time PCR machine. Results were normalized with *β*-actin (t-test: ****P<0.0001; ***P<0.005; **P<0.01; *P<0.05; ns: no significance). All the primers used in the study gave rise to single product of the expected size in agarose gel analysis.

### *In vitro* ubiquitination assays

A 0.5- ml conjugation reaction containing 250 nM Uba1, 600 *μ*M Ub, 500 nM Uev1A, 1 *μ*M Smurf1, 500 nM UbcH5B and 200 nM Smad1 in an ATP cocktail (10 mM Hepes, pH7.5, 5 mM MgCl_2_, 5 mM ATP and 0.6 U/ml inorganic phosphatase) was incubated at 30 °C for 90 min. Reactions were terminated by the addition of TCA to a final concentration of 10% and processed for a 12% SDS-PAGE analysis with antibodies against HA (Santa Cruz, sc-7392). To assess the level of Smad1 ubiquitination, conjugation reactions were conducted as above except that the ATP cocktail does not contain inorganic phosphatase and the reaction was terminated by 5% *β*-mercaptoethanol. The above product was incubated with anti-Flag magic beads (Sigma, M8823) overnight at 4 °C. Protein-bound beads were washed five times with a lysis buffer and eluted in an SDS-PAGE sample buffer. Eluted proteins were analyzed by immunoblotting with rabbit anti-HA antibodies (Bethyl, Montgomery, TX, USA, A190-208 A, 1:5000).

### Significance

Uev1A has been previously shown to promote tumorigenesis and metastasis via the NF-κB and possibly other signaling pathways, while this study identifies *UEV1A* as a putative tumor suppressor in OS cells. Furthermore, all known functions of the Uev family proteins to date are to serve as a cofactor of Ubc13 to promote K63-linked polyubiquitination, while this study shows that the newly identified Uev1A function is independent of Ubc13, which opens a door to Uev family functions beyond the conventional Ubc13 pathway. This study offers a novel means of inducing OS cell differentiation and hence may shed light to the future cure of OS.
