# Supplementary material for: Uev1A facilitates osteosarcoma differentiation by promoting Smurf1-mediated Smad1 ubiquitination and degradation
Source: Cell Death Dis. 2017 Aug 3;8(8):e2974–. doi: 10.1038/cddis.2017.366 (PMC5596555; doi:10.1038/cddis.2017.366)
Supplement: Supplementary Figure Legends [file cddis2017366x1.pdf]

Supplementary figure legends

Figure S1 The expression of Uev1A in the early differentiation stage of OC cells. (**a**) Real-time PCR assay showing mRNA levels of Uev1A and differentiation marker genes on the 3rd day of differentiation. (**b**) Real-time PCR assay showing the mRNA levels of Uev1A and differentiation marker genes on the 7th day of differentiation.

Figure S2 Assessment of *UBC13* and *UEV1A-F38A* overexpression in U2OS cells *in vivo*. (**a**,**b**) *UBC13* promotes the transfer of p65 into the nucleus. *UBC13* or empty vector transfected cells were subjected to immunocytochemistry as described. Only Flag-positive cells were used to score nuclear enrichment of p65. (**a**) Representative images. (**b**) Statistical analysis. At least 30 Flag-positive cells were counted in each view and at least 3 separate views were counted. The results are means ± SD; *** $p < 0.001$. (**c**) The Uev1A-F38A (Uev1Am) mutation abolishes its interaction with Ubc13 *in vivo*. A co-IP assay with an anti-HA antibody for IP and an anti-Ubc13 antibody for WB. (**d**) Relative levels of *UEV1A* and *UEV1Am* expression in U2OS cells.

Figure S3 Effects of *UEV1A* overexpression on the expression of selected proto-oncogenes and stem-cell related genes, and ADM-induced cell death. (**a**) ICC assays to assess the expression of cMyc; (**b**) Real-time PCR assay to measure the expression of OS-related oncogenes in *UEV1A*-overexpressed OS cells. (**c-e**) ICC assays to assess the expression of Aldh1 (**c**). Sox2 (**d**) and Oct4 (**e**). (**f**) Effects of *UEV1A* expression on ADM-induced cell death the in the wild-type and *UEV1A* expression cells. Cells untreated with ADM serve as a control.

Figure S4 Involvement of UbcH5B, Smurf1 and Smad1 in OS cell differentiation. (**a**) Effects of *SMURF1* overexpression on the Smad1 degradation. Experimental conditions were as described in Fig. 4. (**b**) Uev1A interacts with Ubc13 but not UbcH5B. A His_6 pull-down assay was performed to detect direct interaction between UbcH5B or Ubc13 and Uev1A. Experimental conditions were as described in Fig. 5d**.** (**c-h**) Experimental manipulation of cellular levels of *SMURF1*, *UBCH5B* and *SMAD1* alters OC marker gene expression. (**c,d**) Overexpression of *SMURF1*. (**e,f**) Overexpression of *UBCH5B*. (**g,h**) Depletion of *SMAD1*.

Figure S5 Effects of *UEV1A* or *UBCH5B* overexpression on SAOS2 cell differentiation. (**a**) Overexpression of *UEV1A* leads to reduced Smad1 level. (**b**) Overexpression of *UEV1A* or *UEV1Am* alters OS differentiation marker gene expression. (**c**) Overexpression of *UBCH5B* leads to altered differentiation marker gene expression.

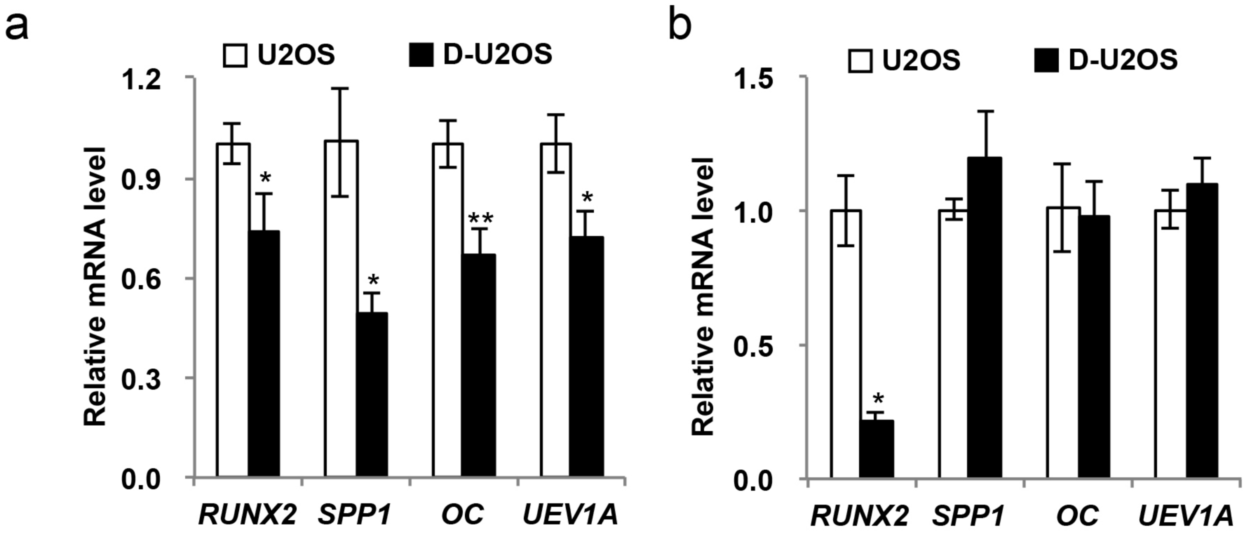
a
U2OS
D-U2OS
Relative mRNA level
1.2
0.9
0.6
0.3
0.0
*
*
**
*
RUNX2
SPP1
OC
UEV1A
b
U2OS
D-U2OS
Relative mRNA level
1.5
1.0
0.5
0.0
*
RUNX2
SPP1
OC
UEV1A

Figure S1

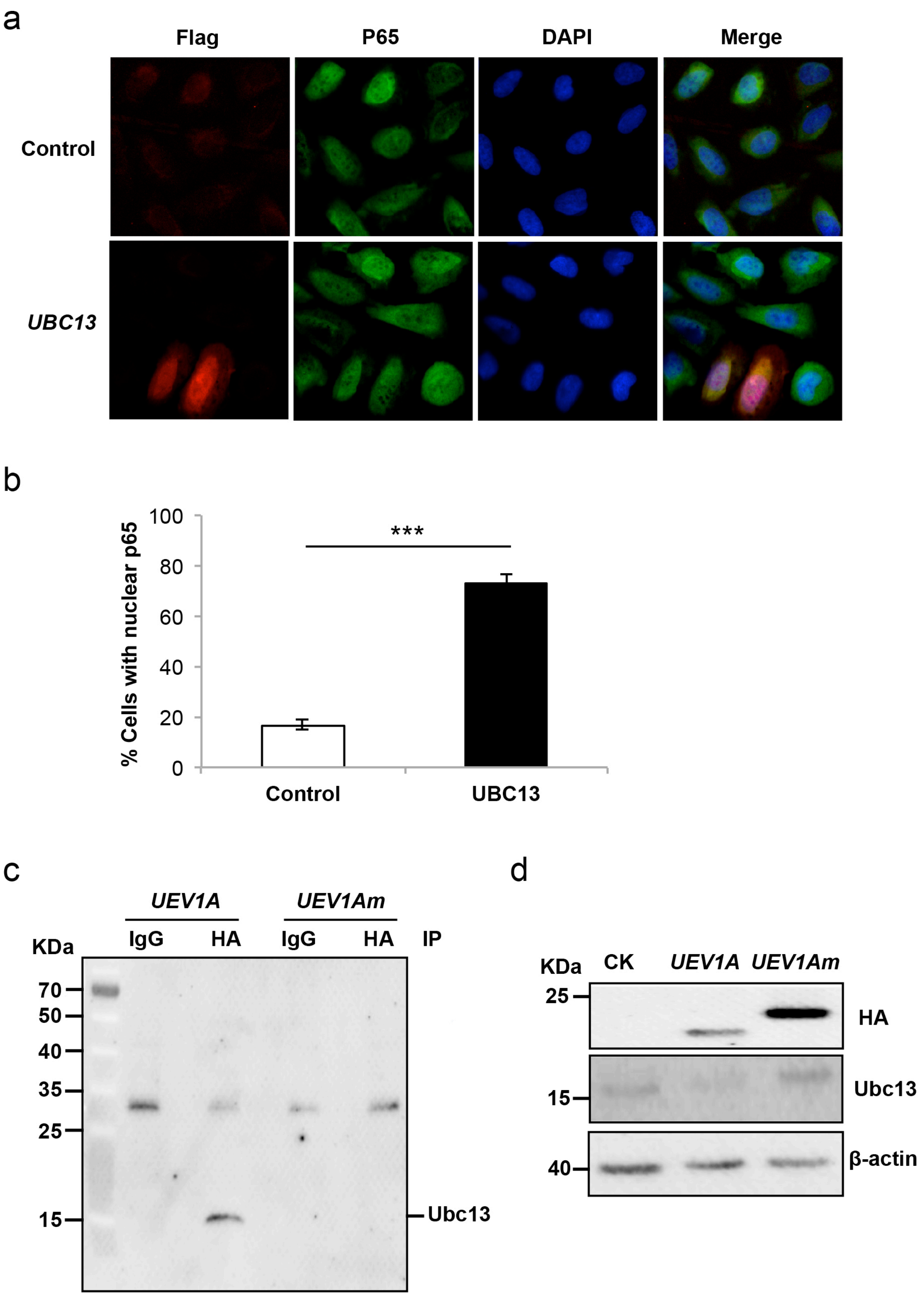
a
Flag
P65
DAPI
Merge
Control
UBC13
b
% Cells with nuclear p65
100
80
60
40
20
0
***
Control
UBC13
c
UEV1A
UEV1Am
IgG
HA
IgG
HA
IP
KDa
70
50
40
35
25
15
Ubc13
d
KDa
CK
UEV1A
UEV1Am
25
HA
Ubc13
15
40
β-actin

Figure S2

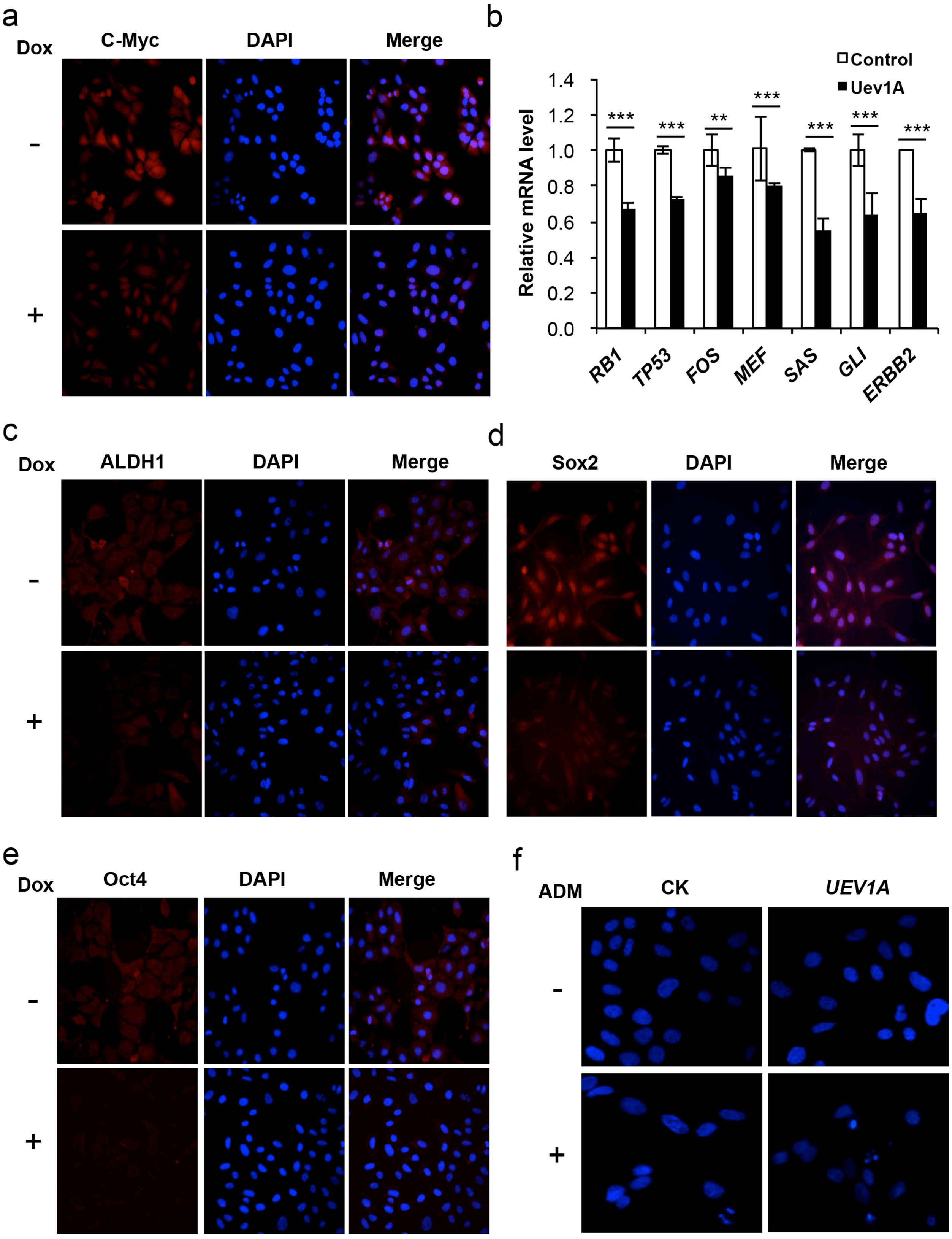
a
Dox
C-Myc
DAPI
Merge
-
+
b
Control
Uev1A
Relative mRNA level
1.4
1.2
1.0
0.8
0.6
0.4
0.2
0.0
***
**
RB1
TP53
FOS
MEF
SAS
GLI
ERBB2
c
Dox
ALDH1
DAPI
Merge
d
Sox2
DAPI
Merge
e
Dox
Oct4
DAPI
Merge
f
ADM
CK
UEV1A

Figure S3

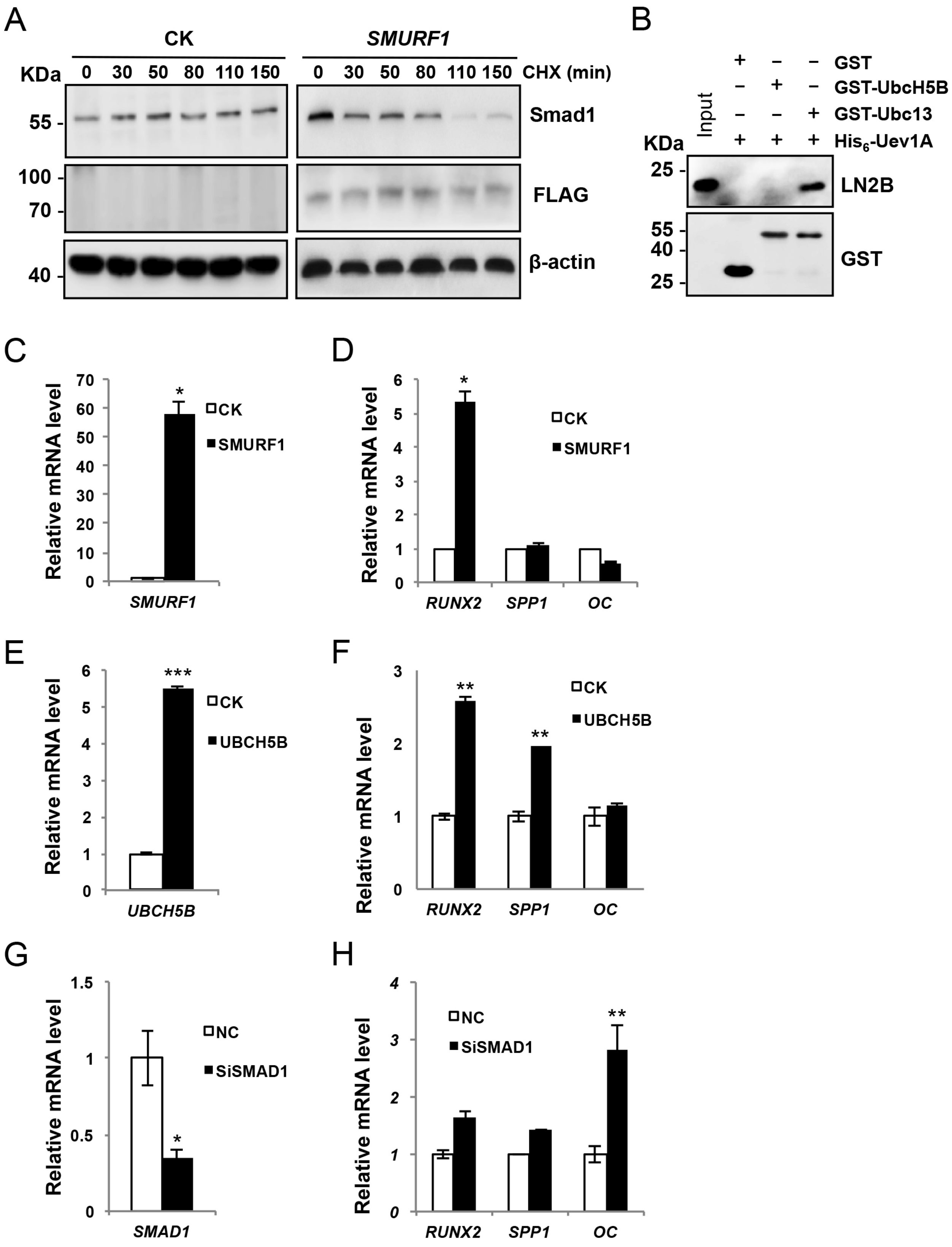
A
CK
SMURF1
KDa
0 30 50 80 110 150
0 30 50 80 110 150 CHX (min)
55
Smad1
100
70
FLAG
40
β-actin
B
Input
+ – – GST
– + – GST-UbcH5B
– – + GST-Ubc13
+ + + His6-Uev1A
KDa
25
LN2B
55
40
25
GST
C
Relative mRNA level
70
60
50
40
30
20
10
0
*
CK
SMURF1
SMURF1
D
Relative mRNA level
6
5
4
3
2
1
0
*
CK
SMURF1
RUNX2
SPP1
OC
E
Relative mRNA level
6
5
4
3
2
1
0
***
CK
UBCH5B
UBCH5B
F
Relative mRNA level
3
2
1
0
**
**
CK
UBCH5B
RUNX2
SPP1
OC
G
Relative mRNA level
1.5
1
0.5
0
*
NC
SiSMAD1
SMAD1
H
Relative mRNA level
4
3
2
1
0
**
NC
SiSMAD1
RUNX2
SPP1
OC

Figure S4

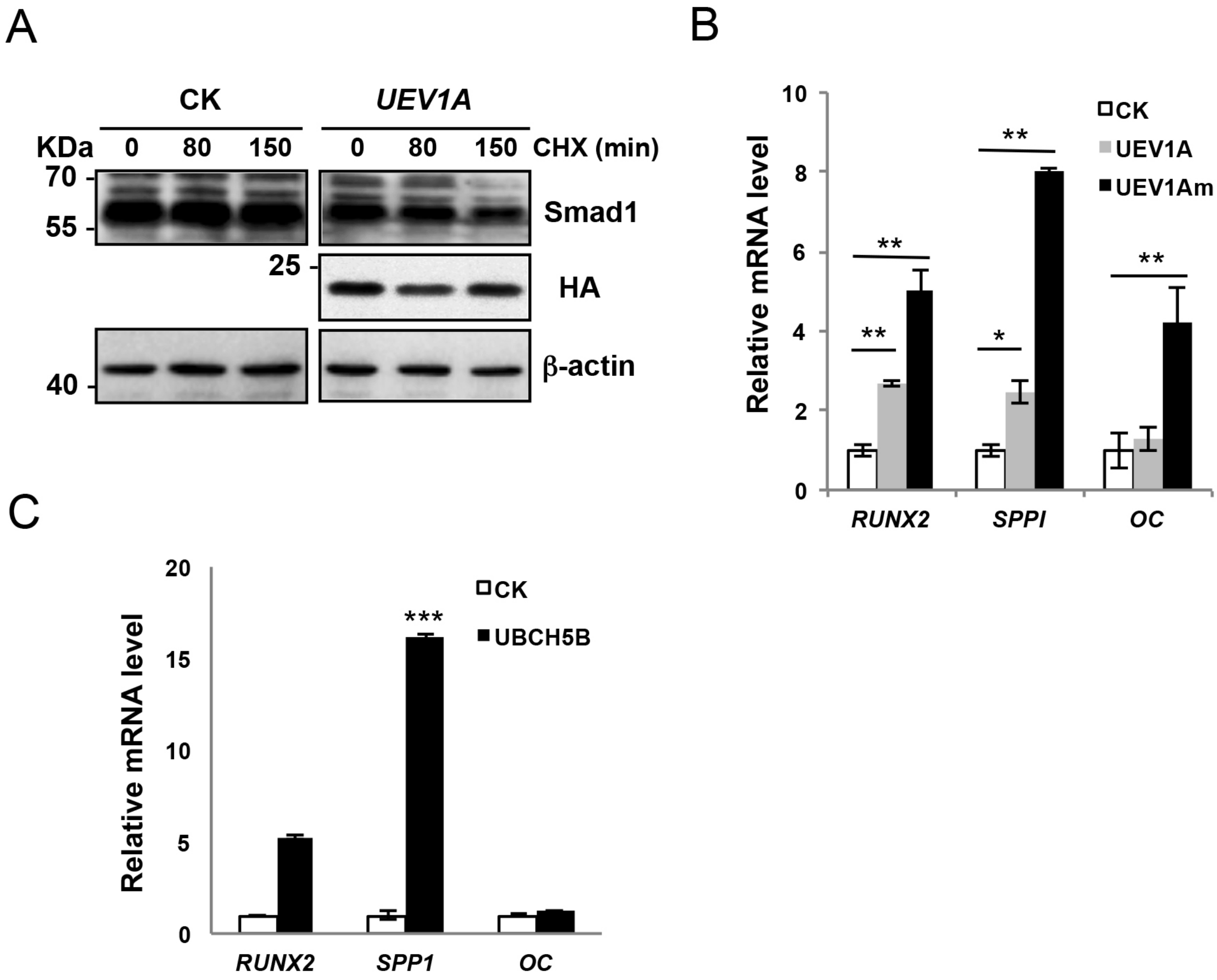
A
CK
UEV1A
KDa
0
80
150
0
80
150
CHX (min)
70
55
Smad1
25
HA
40
β-actin
B
10
8
6
4
2
0
Relative mRNA level
CK
UEV1A
UEV1Am
**
**
*
**
**
RUNX2
SPPI
OC
C
20
15
10
5
0
Relative mRNA level
CK
UBCH5B
***
RUNX2
SPP1
OC

Figure S5
